# Pneumonic-type lung adenocarcinoma with different ranges exhibiting different clinical, imaging, and pathological characteristics

**DOI:** 10.1186/s13244-021-01114-2

**Published:** 2021-11-17

**Authors:** Ji-wen Huo, Xing-tao Huang, Xian Li, Jun-wei Gong, Tian-you Luo, Qi Li

**Affiliations:** 1 Department of Radiology, The First Affiliated Hospital of Chongqing Medical University, No. 1 Youyi Road, Yu zhong District, Chongqing, 400016 China; 2 Department of Pathology, Chongqing Medical University, No.1 Youyi Road, Yuzhong District, Chongqing, 400016 China

**Keywords:** Pneumonic-type lung adenocarcinoma, Tomography (X-ray computed), Epidermal growth factor receptor

## Abstract

**Background:**

Pneumonic-type lung adenocarcinoma (PLADC) with different ranges might exhibit different imaging and clinicopathological features. This study divided PLADC into localized PLADC (L-PLADC) and diffuse PLADC (D-PLADC) based on imaging and aimed to clarify the differences in clinical, imaging, and pathologic characteristics between the two new subtypes.

**Results:**

The data of 131 patients with L-PLADC and 117 patients with D-PLADC who were pathologically confirmed and underwent chest computed tomography (CT) at our institute from December 2014 to December 2020 were retrospectively collected. Patients with L-PLADC were predominantly female, non-smokers, and without respiratory symptoms and elevated white blood cell count and C-reactive protein level, whereas those with D-PLADC were predominantly male, smokers, and had respiratory symptoms and elevated white blood cell count and C-reactive protein level (all *p* < 0.05). Pleural retraction was more common in L-PLADC, whereas interlobular fissure bulging, hypodense sign, air space, CT angiogram sign, coexisting nodules, pleural effusion, and lymphadenopathy were more frequent in D-PLADC (all *p* < 0.001). Among the 129 patients with surgically resected PLADC, the most common histological subtype of L-PLADC was acinar-predominant growth pattern (76.7%, 79/103), whereas that of D-PLADC was invasive mucinous adenocarcinoma (80.8%, 21/26). Among the 136 patients with EGFR mutation status, L-PLADC had a significantly higher EGFR mutation rate than D-PLADC (*p* < 0.001).

**Conclusions:**

L-PLADC and D-PLADC have different clinical, imaging, and pathological characteristics. This new imaging-based classification may help improve our understanding of PLADC and develop personalized treatment plans, with concomitant implications for patient outcomes.

## Key points

- L-PLADC and D-PLADC have different clinical and imaging findings.
- Acinar-predominant growth pattern is the most common histological subtype of L-PLADC.
- Invasive mucinous adenocarcinoma is the most common histological subtype of D-PLADC.
- L-PLADC has a significantly higher EGFR-mutations rate than that of D-PLADC.

## Background

Lung adenocarcinoma (LADC) is the most common histological type of lung cancer [1]. It has a wide variety of manifestations on computed tomography (CT), and sometimes can represent nonobstructive focal or diffuse pulmonary consolidation, which is usually misdiagnosed as pneumonia [2]. This type of LADC is also known as pneumonic-type LADC (PLADC) [3, 4]. Previous studies demonstrated that PLADC corresponds to bronchioloalveolar carcinoma (BAC) with a predominant mucinous histologic subtype, and several investigators have studied the imaging features of PLADC [3–7]. Jung et al. [4] noted that CT findings favored the diagnosis of PLADC based an irregular air bronchogram and a bulging fissure. Aquino et al. [5] reported that CT findings suggestive of PLADC included unresolving peripheral consolidation and coexisting nodules, whereas no significant differences were observed in air bronchogram, leafless tree, bulging fissure, cysts or cavities, low-attenuation consolidation, and CT angiogram sign between pneumonic-type BAC and infectious pneumonia. Im et al. [6] found that CT findings of pneumonic-type BAC included low-attenuation consolidation and CT angiogram sign. Obviously, some of these findings are inconsistent, which may lead to delayed diagnosis and unnecessary biopsies. Moreover, the term consolidation seems to be confusing as the definition of involved range differs among institutions [4–8]. Previous observations of our group in clinical practice showed that PLADC with different ranges might exhibit different imaging and clinicopathological features. Therefore, we speculated that the different outcomes reported in the literature among CT findings of PLADC are owing to the fact that PLADC is a heterogeneous group in which different patterns might be identified based on clinical, radiological, and histopathological findings.

To date, little research has focused on the characteristics of PLADC involving focal or diffuse area of the lung. In this study, we classified PLADC into localized PLADC (L-PLADC) and diffuse PLADC (D-PLADC) based on imaging and aimed to investigate whether clinical, imaging, and pathological characteristics of L-PLADC were different from those of D-PLADC, and if so, we intended to assess these differences.

## Materials and methods

### Patients

The study protocol was approved by the ethics committee of our institute, and the need for informed consent was waived owing to the retrospective nature of the study. A total of 7248 patients with LADC from December 2014 to December 2020 were initially included. Inclusion criteria were as follows: (1) patients who underwent chest CT scan at our institute and (2) those confirmed pathologically by surgical resection, biopsy, and cytology. Subsequently, two experienced radiologists with > 10 years of experience in chest imaging interpreted CT images on a picture archiving and communication system (PACS) workstation (Vue PACS, Carestream) together and classified these patients into three categories: L-PLADC, D-PLADC, and non-PLADC. L-PLADC was defined as localized consolidation involving < 50% of the area of a lobe. D-PLADC was characterized as diffuse consolidation involving ≥ 50% of the area of a lobe or lobes. Non-PLADC refers to tumors that could not be classified as L-PLADC or D-PLADC. Among them, 165 patients were with L-PLADC, 143 with D-PLADC, and 6940 with non-PLADC. Finally, patients with PLADC were further screened according to the following exclusion criteria: (1) patients with a history of chemotherapy, radiotherapy, or other oncologic therapy before CT scans and (2) with an interval of > 1 month between CT imaging and subsequent pathological analysis. In addition, clinical data of all patients, including age, sex, smoking history, respiratory symptoms, laboratory results, diagnostic methods, and clinicopathologic TNM stages at the time of diagnosis, were collected.

### Computed tomography protocols

Chest CT examinations were performed using the Discovery CT750HD (GE Healthcare), Light speed VCT (GE Healthcare), or Somatom Definition Flash (Siemens Healthcare) scanner. All patients underwent CT scanning in the supine position at the end of inspiration during a single breath hold. Imaging parameters were as follows: tube voltage, 120 kVp; tube current, 100–250 mA; and scanning slice thickness/interval, 5 mm/5 mm. A total of 170 patients (170/248, 68.5%) received contrast-enhanced scanning and were injected with nonionic iodinated contrast medium (iohexol 300 mg iodine/mL; Omnipaque, GE Healthcare) at a dosage of 1.5 mL/kg of body weight (total volume: 80–110 mL) using a dual-high-pressure injector via the antecubital vein at a flow rate of 3.0 mL/s. This was followed by injection of 50-mL saline solution. Acquisition times in arterial and delayed phases were triggered at 30 and 120 s, respectively, after the start of contrast medium injection. All images were reconstructed with a section thickness and slice interval of 0.625 mm or 1 mm and 0.625 mm or 1 mm, respectively, for analysis.

### Computed tomography image analysis

The aforementioned radiologists analyzed the CT features of tumors on our PACS workstation, and any disagreements were resolved by discussion until a consensus was reached. The following data were carefully observed and recorded: (1) distribution (single lobe [left upper and lower lobes; right upper, middle, and lower lobes], unilateral multiple lobes, and bilateral multiple lobes); (2) presence of interlobular fissure bulging, hypodense sign (low-density area within consolidation without enhancement), air space (air attenuation in lesions), irregular air bronchogram (air-filled bronchus manifesting as dilatation, rigidity, or narrowing), CT angiogram sign (branching pulmonary vessels extending > 3 cm along a single channel), ground-glass opacity (GGO, increased attenuation without obscuration of underlying lung vessels), coexisting nodules, pleural retraction (one or more linear or cord-like structures connected between the tumor and pleura in the lung window images), pleural effusion, and lymphadenopathy (hilar or mediastinal lymph nodes with a short-axis diameter of > 1 cm). Clinical cancer stages of LADC were evaluated according to the 8th edition of the American Joint Committee on cancer staging system for non-small-cell lung cancer [9].

### Histochemical examination

All selected specimens of PLADC stained with hematoxylin and eosin were analyzed by a pathologist with 12 years of experience in pathological diagnosis of the lung. LADC was determined according to the LADC classification system issued in 2011 [10]. The percentage of each growth pattern (acinar, solid, papillary, micropapillary, lepidic, etc.) in the tumor was semiquantitatively measured in 5% increments, and that with the largest percentage was considered a predominant pattern. Accordingly, the histologic subtype of invasive LADC was described based on its predominant pattern.

### Epidermal growth factor receptor mutation analysis

Surgical specimens, biopsy specimens, and hydrothorax fallen cells of tumors were used for molecular analysis. The epidermal growth factor receptor (EGFR) mutation status (exons 18–21) was examined using a real-time polymerase chain reaction-based amplification refractory mutation system using the Human EGFR Gene Mutations Detection Kit (AmoyDx, Xiamen, China).

### Statistical analysis

Statistical analyses were performed using the International Business Machines Statistical Package for the Social Sciences Statistics for Windows (version 25.0; IBM Corp., Armonk, NY, USA). Single-sample Kolmogorov–Smirnov analysis was used to test the variance in measurement homogeneity. Normally distributed quantitative data are expressed as mean ± standard deviation, and non-normally distributed data are presented as median ± interquartile range, whereas categorical variables were expressed as numbers and percentages. The Chi-square test was employed to compare frequencies of different clinical factors, CT features, and EGFR mutation status between L-PLADC and D-PLADC. A two-tailed *p* value of < 0.05 was considered as statistically significant.

## Results

### Study population

**Fig. 1:**
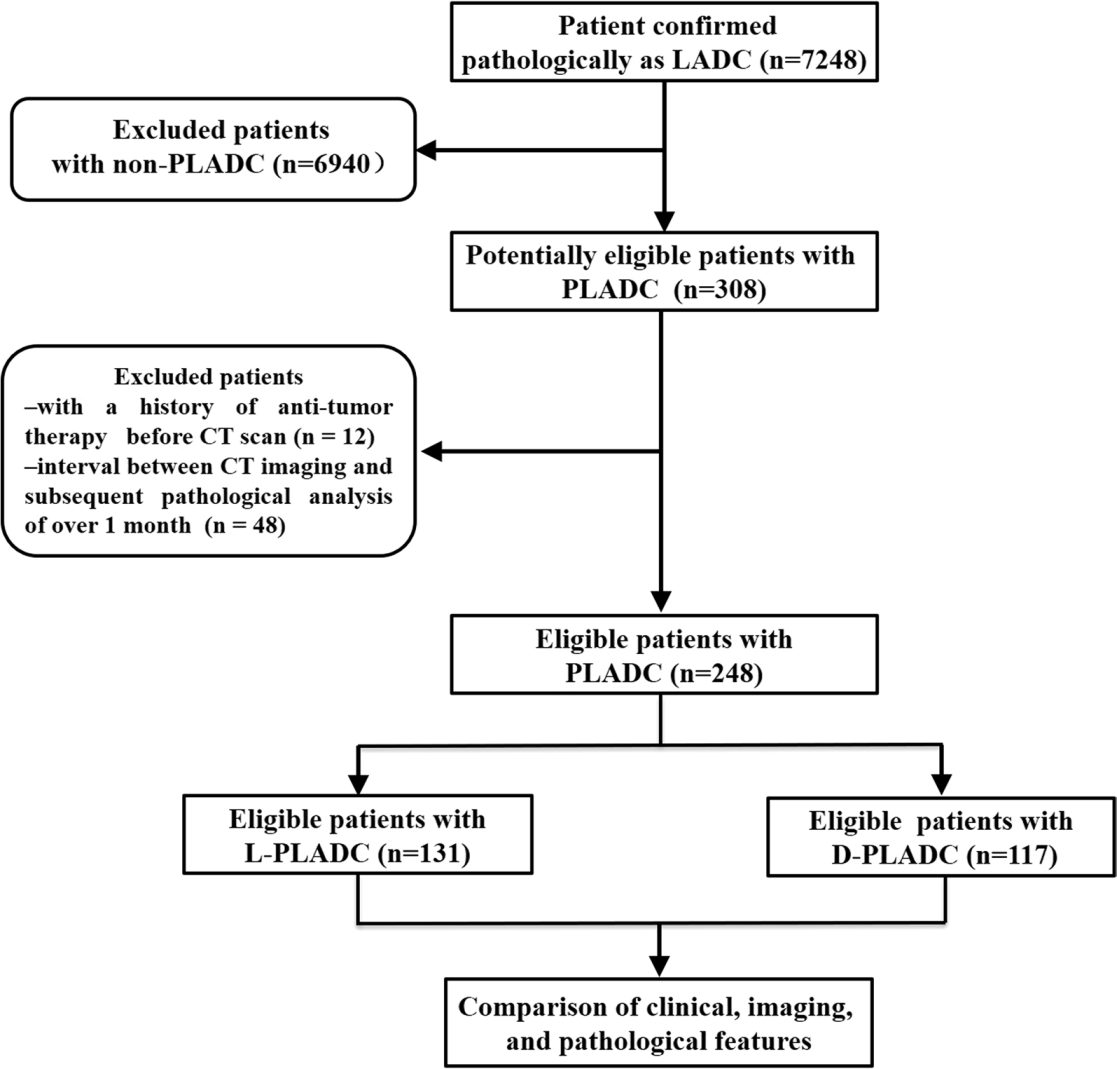
Flow diagram for this study

In total, 308 patients with PLADC were initially selected from 7248 patients who were pathologically confirmed with LADC. Among them, 60 patients were excluded owing to an antitumor therapy history before chest CT scan (*n* = 12) and interval of > 1 month between CT imaging and subsequent pathological analysis (*n* = 48). Finally, 131 patients with L-PLADC and 117 patients with D-PLADC were enrolled for analysis. The flow diagram for this study is shown in Fig. 1. Among the 131 patients with L-PLADC, 108 (82.4%) were confirmed by surgical resection, 14 (10.7%) by bronchoscopy biopsy, six (4.6%) by transthoracic needle biopsy, and three (2.3%) by cytology; 100 patients (76.3%) were in stages I–II and 31 (23.7%) in stages III–IV. Among the 117 patients with D-PLADC, nine (7.7%) were confirmed by surgical resection, 44 (37.6%) by bronchoscopy biopsy, 23 (19.7%) by transthoracic needle biopsy, and 41 (35.0%) by cytology; seven patients (6.0%) were in stages I–II and 110 (94.0%) in stages III–IV.

### Comparison of clinical characteristics between L-PLADC and D-PLADC

Clinical data of patients with L-PLADC and D-PLADC are given in Table 1. The average age of patients with L-PLADC was 62 years (range 35–62 years) and that of patients with D-PLADC was 61 years (range 23–84 years). No significant difference was observed in the age between both groups (*p* > 0.05). C-reactive protein level was assessed in 116 patients who underwent this test. The number of patients in the D-PLADC group who had respiratory symptoms, including cough and sputum, as well as elevated white blood cell count and C-reactive protein level was greater than that of those in the L-PLADC group (all *p* < 0.05). However, no significant differences were observed in symptoms such as fever, blood in sputum, hemoptysis, and chest pain between both groups (all *p* > 0.05).

**Table 1:** Comparison of clinical characteristics between L-PLADC and D-PLADC

Characteristic	Patients with L-PLADC (*n* = 131)	Patients with D-PLADC (*n* = 117)	*p* value
Age (years)			
Means ± standard deviations	62 ± 9	61 ± 11	0.404^a^
Range	35–62	23–84	
Sex			
Male	43/131 (32.8%)	72/117 (61.5%)	< 0.001^b^
Female	88/131 (67.2%)	45/117 (38.5%)	
Smoking			
Non-smokers	97/131 (74.0%)	52/117 (44.4%)	< 0.001^b^
Smokers	34/131 (26.0%)	65/117 (55.6%)	
Respiratory symptoms			
Fever	7/131 (5.3%)	9/117 (7.7%)	0.452^b^
Cough	54/131 (41.2%)	103/117 (88.0%)	< 0.001^b^
Sputum	42/131 (32.1%)	77/117 (65.8%)	< 0.001^b^
Blood in sputum	13/131 (9.9%)	13/117 (11.1%)	0.761^b^
Hemoptysis	8/131 (6.1%)	11/117 (9.4%)	0.330^b^
Chest pain	16/131 (12.2%)	13/117 (11.1%)	0.787^b^
No symptoms	62/131 (47.3%)	5 /117 (4.3%)	< 0.001^b^
Laboratory results			
Elevation of white blood cell count	13/131 (9.9%)	41/117 (35.0%)	0.003^b^
Elevation of C-reactive protein level ^c^	10/32 (31.3%)	66/84 (78.6%)	< 0.001^b^

L-PLADC, localized pneumonic-type lung adenocarcinoma; D-PLADC, diffuse pneumonic-type lung adenocarcinoma

^a^Two independent samples Student’s t test

^b^Chi-squared test

^c^C-reactive protein level was not assessed in 132 patients who did not undergo this test

### Comparison of CT findings between L-PLADC and D-PLADC

**Fig. 2:**
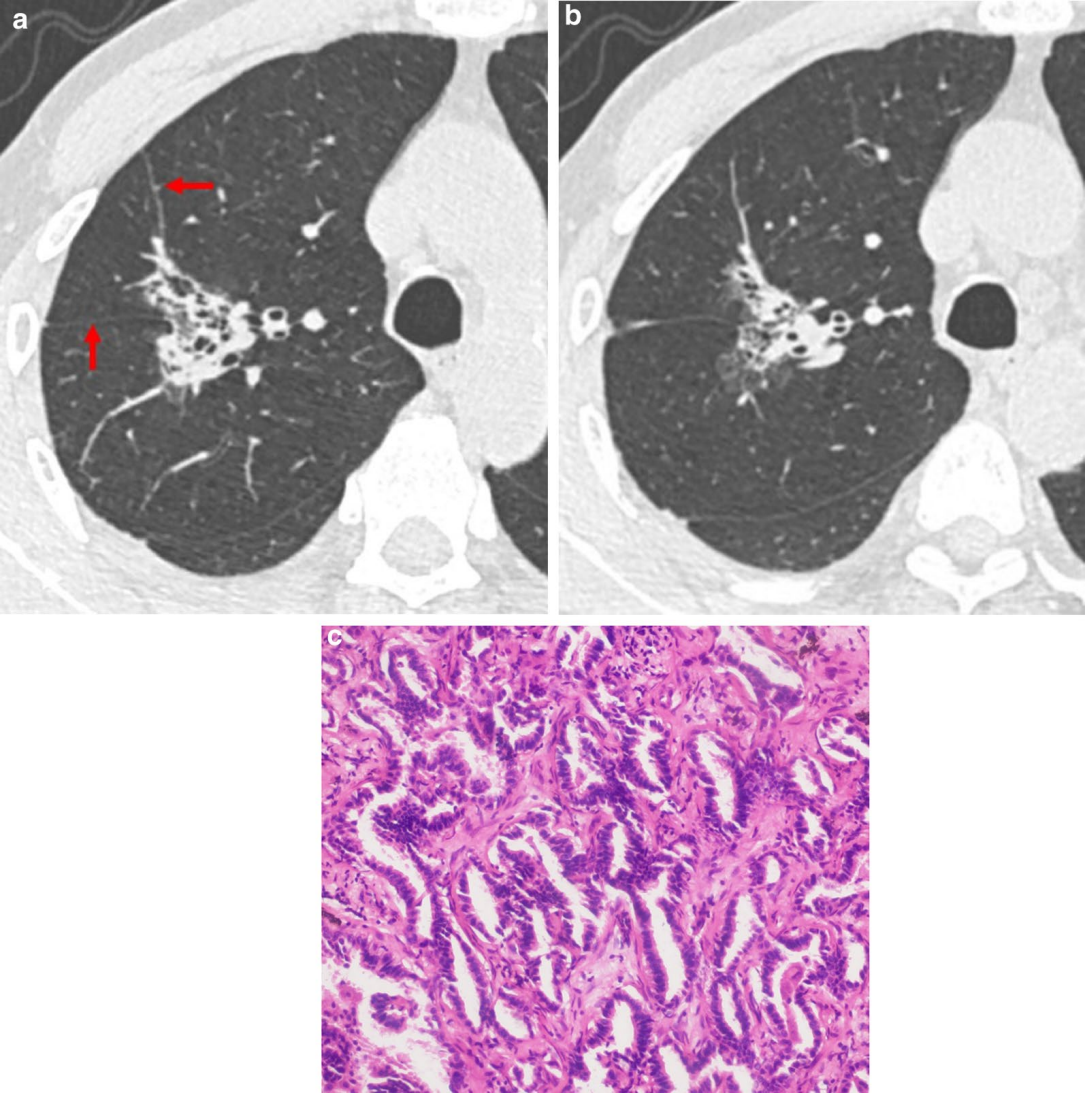
L-PLADC in a 65-year-old man without symptoms. **a**, **b** Axial CT images of the lung window indicate a localized consolidation with irregular air bronchogram, GGO component, and pleural retraction (red arrow) in the right upper lobe. **c** Photomicrograph (hematoxylin and eosin staining, ×200) of surgical specimens confirmed LADC with an acinar-predominant pattern

**Fig. 3:**
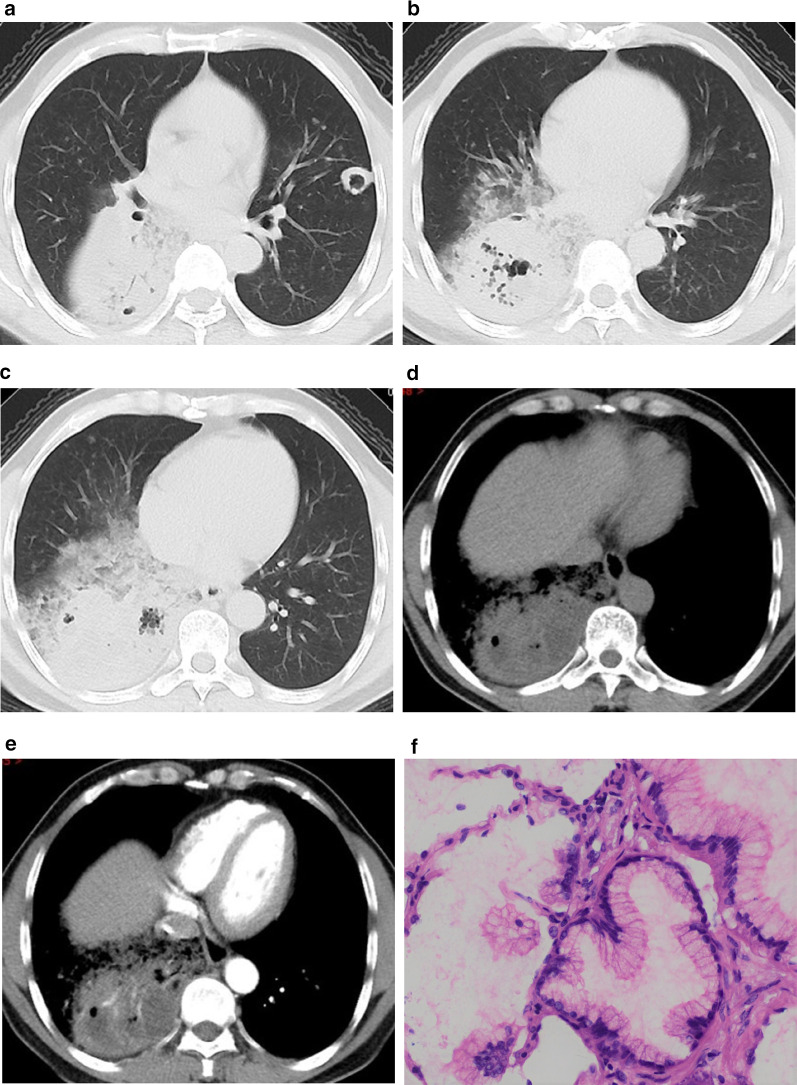
D-PLADC in a 49-year-old man with cough and sputum for 6 months. **a–c** Axial CT images of the lung window indicate diffuse consolidation with interlobular fissure bulging, air space, GGO, irregular air bronchogram, and coexisting nodules in bilateral multiple lobes. **d–e** Axial CT images of the mediastinal window at unenhanced scan (**d**) and arterial phase (**e**) indicate hypodense sign and CT angiogram within consolidation, respectively. **f** Photomicrograph (hematoxylin and eosin staining, ×400) of biopsy specimens confirmed invasive mucinous adenocarcinoma

CT features of patients with L-PLADC and D-PLADC are given in Table 2. Hypodense and CT angiogram signs were assessed in 170 patients who underwent contrast-enhanced scanning. Bilateral multiple lobes distribution was most common in the D-PLADC group. Interlobular fissure bulging, hypodense sign, air space, CT angiogram sign, coexisting nodules, pleural effusion, and lymphadenopathy were more common in patients with D-PLADC, whereas pleural retraction was more frequently seen in patients with L-PLADC (all *p* < 0.001). Both D-PLADC and L-PLADC showed high percentages of irregular air bronchogram and GGO. However, no significant differences were observed in irregular air bronchogram and GGO between both groups (all *p* > 0.05; Figs. 2A, 3A–E).

**Table 2:** Comparison of CT features between L-PLADC and D-PLADC

CT Features	Patients with L-PLADC (*n* = 131)	Patients with D-PLADC (*n* = 117)	*p* value^a^
Distribution			
Single lobe	131/131 (100%)	36/117 (21.1%)	–
Right upper lobe	49/131 (37.4%)	6/117 (5.1%)	–
Right middle lobe	12/131 (9.2%)	0/117 (0%)	–
Right lower lobe	22/131 (16.8%)	14/117 (12.0%)	–
Left upper lobe	35/131 (26.7%)	6/117 (5.1%)	–
Left lower lobe	13/131 (9.9%)	10/117 (8.5%)	–
Right multiple lobes	0/131 (0%)	20/117 (17.1%)	
Left multiple lobes	0/131 (0%)	5/117 (4.3%)	–
Bilateral multiple lobes	0/131 (0%)	56/117 (47.9%)	–
Interlobular fissure bulging	8/131 (6.1%)	48/117 (41.0%)	< 0.001^a^
Hypodense sign ^b^	22/82 (26.8%)	79/88 (89.8%)	< 0.001^a^
Air space	40/131 (30.5%)	68/117 (58.1%)	< 0.001^a^
Irregular air bronchogram	90/131 (68.7%)	81/117 (69.2%)	0.928 ^a^
CT angiogram sign ^b^	22/82 (21.8%)	79 /88 (89.8%)	< 0.001^b^
GGO	101/131 (77.1%)	94/117 (80.3%)	0.534^a^
Coexisting nodules	15/131 (11.5%)	73/117 (62.4%)	< 0.001^a^
Pleural retraction	102/131 (77.9%)	8/117 (6.8%)	< 0.001^a^
Pleural effusion	6/131 (4.6%)	72/117 (61.5%)	< 0.001^a^
Lymphadenopathy	13/131 (9.9%)	53/117 (45.3%)	< 0.001^a^

CT, computed tomography; L-PLADC, localized pneumonic-type lung adenocarcinoma; D-PLADC, diffuse pneumonic-type lung adenocarcinoma; GGO, ground-glass opacity

^a^Chi-squared test

^b^Hypodense sign and CT angiogram sign was not assessed in 78 patients who did not undergo contrast-enhanced scanning

### Comparison of histological subtypes between L-PLADC and D-PLADC

**Fig. 4:**
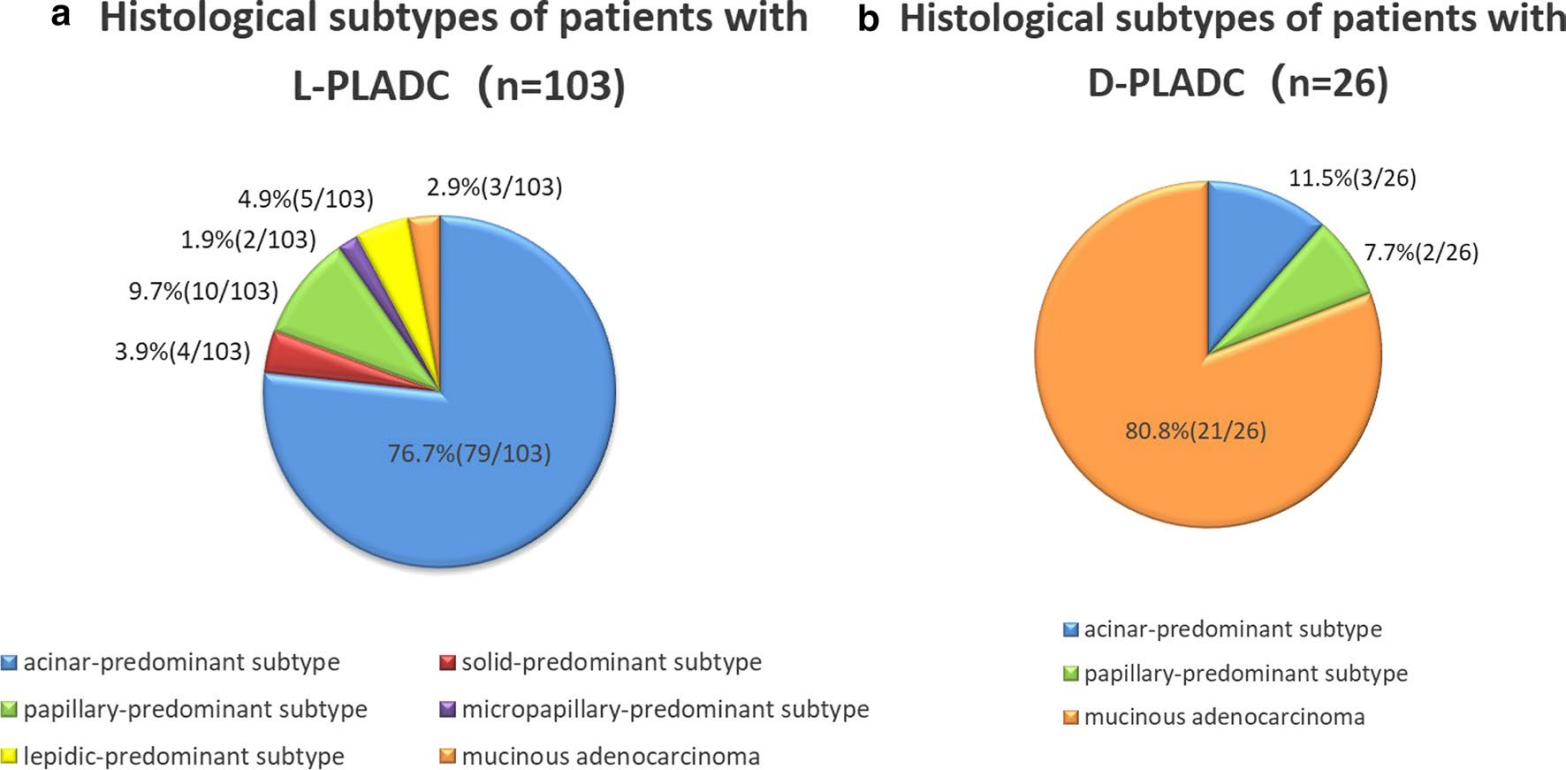
Distribution diagrams for histological subtypes of patients with L-PLADC and D-PLADC. *Notes*: Data are presented as %

Histological subtypes of patients with L-PLADC and D-PLADC are shown in Fig. 4. In total, 103 patients with L-PLADC and 26 patients with D-PLADC were confirmed as invasive LADC with identifiable growth patterns. Acinar-predominant growth pattern was found to be the most common histological subtype for patients with L-PLADC (76.7%, 79/103), followed by papillary-predominant growth pattern (9.7%, 10/103) and lepidic-predominant growth pattern (4.9%, 5/103) (Figs. 2C, 4A). Invasive mucinous adenocarcinoma was the most common subtype in patients with D-PLADC (80.8%, 21/26), followed by acinar-predominant growth pattern (11.5%, 3/26) and papillary-predominant growth pattern (7.7%, 2/26) (Figs. 3F, 4B).

### EGFR mutation analysis

A total of 136 patients with PLADC underwent molecular analyses of EGFR mutation. Among them, 64 patients had L-PLADC and 76.6% (49/64) were positive for EGFR mutations, including 22 patients harboring a single exon 19 deletion mutation, 21 harboring a single L858R mutation, and six harboring other mutation subtypes; 72 patients had D-PLADC and 38.9% (28/72) were positive for EGFR mutations, including 18 patients harboring a single 19 exon deletion mutation, four harboring a single L858R mutation, and six harboring other mutation subtypes. EGFR mutation rate of L-PLADC was significantly higher than that of D-PLADC (*p* < 0.001).

## Discussion

This study aimed to investigate whether it is reasonable to divide PLADC into two new subtypes according to different ranges of lung involvement. To our knowledge, this is the first study to focus on differences in clinical, imaging, and pathological features between D-PLADC and L-PLADC. Several major findings were noted in this study.

First, we found that patients with L-PLADC were predominantly women and non-smokers, whereas those with D-PLADC were predominantly men and smokers. Studies have indicated that a high proportion of patients with LADC are women and non-smokers [11, 12], which is consistent with the demographic characteristics of patients with L-PLADC, but contradicts with those of patients with D-PLADC. Considering that L-PLADC and D-PLADC are new imaging-based subtypes, few studies have focused on their differential clinical features. Future studies with larger sample sizes are warranted to substantiate our findings. Furthermore, our results showed that the number of patients with L-PLADC without  respiratory symptoms and elevated white blood cell count and C-reactive protein level was higher than those with D-PLADC without these symptoms. Chu et al. [13] indicated that the number of patients with lung cancers who were asymptomatic was higher than that of those with inflammatory lesions. Yao et al. [14] have showed that the C-reactive protein is a sensitive and nonspecific inflammatory marker, and its increased level is highly indicative of infection. Hence, these clinical characteristics of L-PLADC are similar to those reported in previous studies about lung cancer; however, some features of D-PLADC are contrary to those reported in the literature and often overlap with pulmonary infection, which may result in a diagnostic error [13, 14]. Familiarity with these special clinical features of D-PLADC may contribute to its early diagnosis. We speculated that involvement of more lobes leads to a higher frequency of respiratory symptoms in patients with D-PLADC. Our results showed that a higher number of  patients with D-PLADC were in stages III–IV and had pleural effusion and lymphadenopathy, which were suggestive of a poor prognosis. Previous studies have demonstrated that an inflammatory reaction is closely related to the development and progression of malignant tumors, and increase in some inflammatory indexes such as C-reactive protein and neutrophils is associated with poor prognosis of tumors [15, 16]. This may be a good explanation for the findings of the present study.

Next, we found that imaging features were diverse between L-PLADC and D-PLADC. The present study showed that pleural retraction was closely related to L-PLADC, whereas interlobular fissure bulging, hypodense sign, air space, CT angiogram sign, and coexisting nodules were closely correlated with D-PLADC. Possible causes underlying these findings could likely be attributed to the histological subtype differences between L-PLADC and D-PLADC. According to our results, L-PLADC showed a high frequency of acinar-predominant subtype, whereas D-PLADC had a high occurrence of invasive mucinous adenocarcinoma. Pleural retraction is indicative of tumor contraction resulted from the narrowing or collapse of alveolar spaces or from fibrotic areas in tumors, which is generally observed in patients with non-mucinous adenocarcinoma [17]. Generally, invasive mucinous adenocarcinoma contains columnar or goblet cells with abundant intracellular or extracellular mucus admixed with invasive adenocarcinoma patterns with stromal invasion. Tumor cells usually grow along the alveolar wall and secrete abundant mucin, thereby filling the alveolar lumina, which may cause gelatinous parenchymal consolidation and increased volume of the involved lung parenchyma [3, 18], contributing to interlobular fissure bulging and hypodense sign. After intravenous injection of contrast medium, the consolidated lung typically manifests as an area of low attenuation on CT, allowing an enhanced branching of pulmonary vessels to be identified as CT angiogram sign. The presence of air space may be false in that tumor cell invades the bronchus and forms a unidirectional check-valve, which may allow air in but not out, resulting in the formation of a pseudo-cavity [3, 7, 19, 20]. The coexisting nodules may be associated with multicentric growth or metastasis of tumors [21]. Consistent with our findings, some investigators showed that interlobular fissure bulging, low attenuation within consolidation, air space, CT angiogram sign, and coexisting nodules are strongly suggestive of PLADC [3–8, 17–20]. Our results also indicated that irregular air bronchogram and GGO were frequently detected in both subtypes of PLADC, which conforms to the results of some investigators [4, 7, 22]. Irregular air bronchogram may be associated with tumor invasion or desmoplastic reaction [22], whereas GGO may correspond to incomplete filling of the alveolar spaces and slight thickening of the alveolar wall owing to infiltration or mucus secretion by tumor cells [23]. Additionally, pleural effusion and lymphadenopathy were found to be more common in D-PLADC than L-PLADC, indicating a poor prognosis in patients with D-PLADC; this result is consistent with the results of other scholars, who stated that the pneumonic type of lung invasive mucinous adenocarcinoma correlated with poor outcome [24–26]. Therefore, this new imaging-based typing of PLADC may be helpful for us to better understand its CT findings and predict its prognosis.

Furthermore, our findings showed that PLADC with focal and diffuse consolidation had different histological subtypes and EGFR mutation status. Our results indicated that L-PLADC showed a strong tendency for acinar-predominant subtype and EGFR positive mutations, whereas D-PLADC was closely related to invasive mucinous adenocarcinoma and EGFR negative mutations. Liu et al. [27] evaluated the clinical–radiological–pathological characteristics and prognosis of advanced pneumonic-type lung cancer, which predominantly involved diffuse area of lung, and found that the majority of patients had invasive mucinous adenocarcinoma and correlated with the absence of EGFR mutations. Liu et al. [8] investigated the CT features that correlated with EGFR mutations in surgically resected pneumonic-type lung cancer, which predominantly involved the focal area of lung, and found that most patients were with acinar-predominant invasive adenocarcinoma and correlated with the presence of EGFR mutations. Our results were in agreement with previous results. Some scholars demonstrated that EGFR mutations correlated with women and non-smokers [28, 29]. A prior study conducted by Yanagawa et al. [30] revealed that EGFR mutations had high frequency among some histological subtypes of adenocarcinoma, including adenocarcinoma in situ (62%), minimally invasive adenocarcinoma (60%), lepidic (77%), papillary (50%), acinar (49%), and micropapillary (43%), but a relatively low frequency in solid (28%) and invasive mucinous adenocarcinoma (0%). Therefore, the relatively high expression of EGFR mutations in patients with L-PLADC can be explained by the following: the majority of patients were female and non-smokers; most patients were with adenocarcinoma and had acinar, papillary, and lepidic growth pattern. The presence of EGFR mutations has been clearly proved as a strong point for predicting a good response to EGFR-tyrosine kinase inhibitors [31]. According to our results, patients with L-PLADC in advanced stages may be more likely to benefit from targeted therapy. In contrast, standard chemotherapy may still be a preferred treatment strategy for patients with D-PLADC, and targeted therapy can be given to patients with EGFR-activating mutation.

This study has several limitations that should be considered. First, the retrospective nature of this study might have resulted in selection bias; further, we only performed univariate analyses. Second, only a small number of patients with D-PLADC underwent surgical resection; thus, future studies on the histological subtypes of D-PLADC with larger sample sizes are warranted to substantiate our findings. Third, our analyses were limited to adenocarcinomas and did not address other histologic types. This was done because the majority of PLADC is found in adenocarcinomas.

## Conclusion

In conclusion, our findings demonstrate that L-PLADC and D-PLADC have different clinical, imaging, and pathological characteristics. This new imaging-based classification may help improve our understanding of PLADC and develop personalized treatment plans, with concomitant implications for patient outcomes.

## Data Availability

The datasets used and/or analyzed during the current study are available from the corresponding author on reasonable request.

## Abbreviations

BAC: Bronchioloalveolar carcinoma
CT: Computed tomography
D-PLADC: Diffuse pneumonic-type lung adenocarcinoma
EGFR: Epidermal growth factor receptor
GGO: Ground-glass opacity
LADC: Lung adenocarcinoma
L-PLADC: Localized pneumonic-type lung adenocarcinoma
PACS: Picture archiving and communication system
PLADC: Pneumonic-type lung adenocarcinoma
